# Curcumin Targets Crispld2 to Suppress Hepatic Stellate Cell Activation via PI3K/AKT Pathway Inhibition in Hepatic Fibrosis

**DOI:** 10.1111/liv.70696

**Published:** 2026-05-13

**Authors:** Ling Liu, Jintao Zheng, Ye Wang, Chenao Yang, Yuqiang Shan, Changku Jia

**Affiliations:** ^1^ Department of Hepatobiliary and Pancreatic Surgery Hangzhou First People's Hospital Affiliated to Medical School of Westlake University Zhejiang Shangcheng District, Hangzhou China; ^2^ Department of Hepatobiliary and Pancreatic Surgery The Fourth School of Clinical Medicine, Zhejiang Chinese Medical University Zhejiang Binjiang District, Hangzhou China; ^3^ Department of Gastroenterological Surgery Hangzhou First People's Hospital Affiliated to Medical School of Westlake University Zhejiang Shangcheng District, Hangzhou China; ^4^ Department of Hepatobiliary and Pancreatic Surgery, the Second Affiliated Hospital School of Medicine, Zhejiang University Zhejiang Shangcheng District, Hangzhou China

**Keywords:** crispld2, curcumin, hepatic fibrosis, hepatic stellate cells, PI3K/AKT pathway

## Abstract

**Background & Aims:**

Hepatic fibrosis (HF) is a key pathological process in the development of chronic liver disease, and the activation of hepatic stellate cells (HSCs) is its core driving factor. Although curcumin, as a natural polyphenolic compound, has therapeutic potential, its specific mechanism in HF is still unclear.

**Methods:**

This study used the CCl4‐induced mouse HF model and explored its mechanism through single‐cell RNA sequencing (scRNA‐seq) combined with in vitro and in vivo experimental systems.

**Results:**

The results showed that curcumin attenuated liver injury and fibrosis levels in mice with HF in a dose‐dependent way, alleviated liver pathological damage, improved liver function indicators, and inhibited the release of inflammatory factors. ScRNA‐seq analysis revealed a significant reduction in the number of activated HSCs after curcumin treatment, and the key gene Crispld2 was identified through perturbation model prediction and pseudo‐temporal analysis. Curcumin significantly downregulated the expression of Crispld2 and inhibited the activation of the PI3K/AKT signalling pathway. In the TGF—β—induced LX‐2 cell activation model, curcumin inhibited HSC proliferation, promoted apoptosis, reduced fibrosis‐related protein expression, and decreased inflammatory cytokine secretion by regulating Crispld2. Overexpression of Crispld2 reversed the anti‐fibrotic effect of curcumin, while the PI3K/AKT pathway inhibitor LY294002 restored its therapeutic effect. Animal experiments further confirmed that curcumin improved HF by regulating the Crispld2/PI3K/AKT axis.

**Conclusions:**

This study elucidates the molecular mechanism by which curcumin exerts anti‐HF effects by inhibiting the activation of the PI3K/AKT pathway mediated by Crispld2, providing a new strategy for targeted therapy of Crispld2.

## Lay Summary

- This study investigated the ameliorative effects of curcumin, a natural compound, on hepatic injury and fibrosis in mouse models of liver fibrosis. The underlying mechanism involves the suppression of the key gene Crispld2, which subsequently blocks the aberrant activation of the PI3K/AKT signalling pathway, thereby inhibiting the activation of hepatic stellate cells and exerting hepatoprotective functions. These findings furnish novel scientific evidence supporting the therapeutic application of curcumin in patients with liver fibrosis and shed new light on the development of Crispld2‐targeted pharmaceutical agents.
- Curcumin can alleviate liver injury and fibrosis in mice with hepatic fibrosis. The underlying mechanism is that curcumin blocks the PI3K/AKT signalling pathway by targeting and inhibiting Crispld2, thereby suppressing the activation of HSCs and ameliorating hepatic fibrosis.

**Abbreviations**
aHSCs: activated hepatic stellate cells
ALB: albumin
ALT: alanine aminotransferase
AST: aspartate aminotransferase
DCs: dendritic cells
ECM: extracellular matrix
Endos: endothelial cells
HF: hepatic fibrosis
HSCs: hepatic stellate cells
HVGs: highly variable genes
IHC: immunohistochemistry
MMPs: matrix metalloproteinases
OD: optical density
PI3K/AKT: phosphatidylinositol 3‐kinase/AKT
scRNA‐seq: single‐cell RNA sequencing
TBIL: total bilirubin
WB: Western blot

## 1 Introduction

Hepatic fibrosis (HF) arises from sustained liver damage triggered by diverse causes. Over time, this fibrotic response disrupts hepatic architecture and performance, leading to cirrhosis and eventual organ failure [1, 2]. Central to this trajectory is the overproduction of extracellular matrix (ECM) by activated hepatic stellate cells (aHSCs) [3]. In healthy livers, HSCs stay quiescent and store abundant vitamin A lipid droplets [4]. When the liver is injured by viruses, alcohol, metabolic stress, or drugs, HSCs switch from a resting state to activated, myofibroblast‐like cells that release abundant α‐SMA, type I collagen, and other ECM components, ultimately forming irreversible fibrotic scars [5]. Consequently, eliminating aHSCs is regarded as a rational anti‐fibrotic strategy, and revealing the molecular mechanism underlying HSC activation is now a critical, still‐unfilled requirement for developing safer and more effective therapies targeting HSCs.

The PI3K/AKT axis is a central signalling hub that regulates cell proliferation, survival, and metabolism. In the liver, it is engaged by growth factors, cytokines, and oxidative stress [6]. A large body of work has established that the hyper‐activation of PI3K/AKT promotes HSC proliferation, suppresses pro‐apoptotic proteins, and thereby prolongs the survival of HSCs [7]. Moreover, the activation of PI3K/AKT signalling up‐regulates ECM‐synthetic proteins, such as fibronectin and laminin, while down‐regulating matrix metalloproteinases (MMPs), thereby decreasing the degradation of ECM [8]. PI3K/AKT signalling also triggers the mTOR or NF‐κB cascades, which boost pro‐inflammatory cytokine release and immune cell infiltration, ultimately amplifying hepatic injury and fibrosis [9, 10]. Thus, the hyper‐activated PI3K/AKT pathway has emerged as a key driver of HF progression, underscoring the imperative for therapeutic strategies that directly target this signalling axis.

Curcumin, a polyphenol found in *Curcuma longa* L., possesses broad anti‐oxidant, anti‐angiogenic and anti‐inflammatory properties that are beneficial in diabetes, cardiovascular disorders, and multiple cancers [11, 12, 13]. Mounting evidence indicates that curcumin also opposes fibrosis. It restrains HSC proliferation and induces apoptosis of aHSCs by modulating autophagy and metabolic reprogramming [14, 15]. Additionally, it also suppresses pathological angiogenesis in the liver by influencing the release of pro‐inflammatory or fibrotic mediators such as PDGF and TGF‐β from ECM while simultaneously regulating MMP activity to oppose fibrosis [16, 17]. Notably, curcumin has been reported to restrain oxidative‐stress‐driven inflammation via PI3K/AKT blockade, thereby alleviating acute liver failure [18]. However, whether curcumin can exert therapeutic effects in HF by modulating PI3K/AKT signalling remains undefined.

Although previous studies have suggested that curcumin may exert anti‐fibrotic effects by inhibiting the PI3K/AKT pathway, its specific molecular targets and precise regulatory mechanisms on HSC activation are still unclear, severely limiting its clinical translation and precise application. In view of this, the aim of this study is to systematically elucidate the molecular mechanism of curcumin in anti‐HF through single‐cell RNA sequencing (scRNA‐seq) combined with in vitro and in vivo functional experiments, identify its key targets, and promote the development of anti‐fibrotic drugs derived from natural products.

## 2 Materials and Methods

### 2.1 Animal Model Construction

This study used 40 4‐ to 6‐week‐old C57BL/6J mice purchased from Shanghai Model Organisms (China). All animal protocols were approved by the Animal Research Ethics Committee of the Second Affiliated Hospital, School of Medicine, Zhejiang University in China (No. 2025–273) and were conducted in strict accordance with the approved guidelines. All animal experiments were performed at the Medical Animal Centre, the Second Affiliated Hospital, School of Medicine, Zhejiang University. After a 1‐week acclimatisation at 22°C, 45%–50% humidity and a 12 h light/12 h dark cycle, mice were randomised into control and model groups. Control mice (*n* = 10) received normal saline (gavage, once daily, 6 weeks) and peanut oil (subcutaneous injection, twice weekly, 15 weeks). Model mice were injected intraperitoneally with a 1:1 mixture of CCl_4_ and peanut oil (twice weekly, 8 weeks) to induce HF. The model mice were further randomised into five sub‐groups: Group 1 (Model group, *n* = 10) received no intervention. Group 2 and Group 3 (Treatment groups, *n* = 5) were administered intragastrically curcumin (MedChemExpress, MCE, USA) at 25 mg/kg or 50 mg/kg, respectively. Group 4 (Cur + oe‐NC, *n* = 5) was transduced with oe‐NC lentivirus and simultaneously received curcumin at 50 mg/kg via gavage. Group 5 (Cur + oe‐Crispld2, *n* = 5) received oe‐Crispld2 lentiviral particles and 50 mg/kg curcumin intragastric administration. Curcumin treatments were administered daily for 6 consecutive weeks. Lentivirus at a titre of 2.5 × 10^7^ pfu/g was delivered via tail‐vein injection every two weeks. Upon completion of treatment, animals were anaesthetised by inhalation of isoflurane (MCE, USA) (induction: 3%–4% in 100% oxygen; maintenance: 1.5%–2% in 100% oxygen). Following the achievement of deep anaesthesia, euthanasia was performed by cervical dislocation. Blood was collected and centrifuged. Centrifuged blood was stored at −20°C, and serum of liver tissues was kept at −80°C for subsequent analyses.

### 2.2 Cell Culture

We cultured human HSC line LX‐2 (RRID: CVCL_5792; BNCC337957, BNCC, China) in RPMI‐1640 complete medium (BNCC, China) containing 10% FBS (Beyotime, China) and human embryonic kidney 293 T cells (RRID: CVCL_0063; BNCC353535, BNCC, China) in DMEM‐H complete medium (BNCC, China) containing 10% FBS and 2 mM L‐glutamine (MCE, USA). All culture media were also supplemented with 1% penicillin–streptomycin (Beyotime, China). Cells were incubated at 37°C with 5% CO_2_.

To induce aHSCs, LX‐2 cells underwent TGF‐β1 exposure (10 ng/mL; MCE, USA). Curcumin was administered at 50 mg/L for treatment. Following a previous study [19], LY294002 (MCE, USA) was dissolved in DMSO to a 10 mM stock, aliquoted, and stored at −20°C. For use, it was diluted to 20 μM in complete medium. Cells were pretreated for 1 h in fresh medium containing LY294002 or an equal volume of DMSO before the experiment.

### 2.3 Cell Transfection

Before transfection, cells were seeded in antibiotic‐free medium and grown to 70%–90% confluence. LX‐2 cells were transfected with oe‐Crispld2, sh‐Crispld2, or the corresponding controls (RiboBio, China) using Lipofectamine 2000 (Thermo Fisher, USA). Transfection efficiency was assessed 48 h later.

For lentiviral vector construction, the Crispld2‐specific cDNA was cloned into the pLVX‐Puro vector (Chemical Book, China), then co‐transfected with packaging vectors psPAX2 and pMD2.G (Chemical Book, China) into 293 T cells with Lipofectamine 2000. Supernatants containing the virus were harvested 48 h post‐transfection.

### 2.4 Histopathological Analysis

The study also examined the pathological injury and fibrotic changes of liver tissues. Samples were firstly fixed in 4% paraformaldehyde (Beyotime, China) for 24 h, then dehydrated, embedded in paraffin, and cut into 5 μm sections. After H&E, Masson and Sirius‐Red (Solarbio, China) staining, slides were examined under a microscope (Olympus, Japan).

### 2.5 Biochemical Assays

We used commercial kits from Nanjing Jiancheng (China) to assess serum albumin (ALB), alanine aminotransferase (ALT), and total bilirubin (TBIL), and kits from Shanghai MLBio (China) to measure aspartate aminotransferase (AST). Absorbance was read on a SpectraMax microplate spectrophotometer (Molecular Devices, USA) or a microplate reader (Thermo Fisher, USA). Each parameter was calculated from the obtained optical density (OD) values.

### 2.6 Elisa

Mouse or human ELISA kits (Shanghai MLBio, China) were used to measure TNF‐α and IL‐6 levels in serum or HSCs following the manufacturer's protocols. OD_450 nm_ was recorded on a microplate reader (Thermo Fisher, USA) to calculate the content of cytokines.

### 2.7 Immunohistochemistry (IHC)

After deparaffinisation and rehydration, liver slices underwent antigen retrieval in pH 6.0 citrate buffer (Aladdin, China). Slides were first treated with 3% H_2_O_2_ (Beyotime) to inactivate endogenous peroxidase, then coated with 1% BSA (Beyotime) to block non‐specific proteins. Primary antibodies were applied overnight, followed by incubation with HRP‐conjugated secondary antibodies at 37°C for 1–2 h. Colour was developed with DAB (Beyotime, China) and photographed under an Olympus microscope (Japan). Antibody details are provided in Table S1.

### 2.8 Single‐Cell RNA Sequencing (scRNA‐Seq)

Liver tissues from the model group and fibrotic mice treated with 50 mg/kg curcumin were harvested and immediately sent to High Precision (Hangzhou) Life Technology Co. Ltd. for scRNA‐seq. Libraries were cleaned twice with SPRI beads and quantified, then subjected to PE150 sequencing on an Illumina NovaSeq 6000 platform. Quality control was performed on the single‐cell data using the “scanpy” package (v1.10.4) in Python. Doublets were removed using the “scrublet” method. Cells were filtered out if mitochondrial gene percentage > 20%, ribosomal gene percentage > 30%, nUMIs < 25 000 or detected genes < 5000. Gene expression counts were normalised, and the top 2 000 highly variable genes (HVGs) were selected on the basis of dispersion. These top 2000 HVGs were scaled and subjected to dimensionality reduction for principal‐component analysis. Five batch‐correction algorithms, i.e., Harmony, scVI, scANVI, BBKNN and Scanorama, were evaluated with the “scIB” Python package (v1.1.5). Harmony was finally selected. The first 30 principal components were supplied to the Leiden algorithm for clustering single‐cell data (resolution = 0.45). The resulting clusters were visualised with UMAP.

### 2.9 Perturbation‐Target Prediction

To quantify which cell types and genes were most affected by curcumin, we created an “Augur” object with the “pertpy” Python package (v0.9.4), using the “random_forest_classifier” as the estimator. Augur leverages machine‐learning models to measure how distinctly stimulated and unstimulated cells partition in high‐dimensional transcriptomic space, thereby assessing the impact of the perturbation on individual cell types and ranking the importance of signature genes.

### 2.10 Pseudotime Analysis

To reveal the activation process of HSCs, the “CytoTRACE” R package (v0.3.3) was applied to compute cell stemness scores. CytoTRACE assigns each cell a score between 0 and 1, with higher scores indicating greater stemness and lower differentiation. The “monocle” package (v2.32.0) in R was then used to reconstruct differentiation trajectories of HSCs. Trajectories were visualised with the “plot_cell_trajectory” function.

### 2.11 KEGG Enrichment Analysis

To explore the differences in the biological characteristics of different cell clusters between the control and the treatment groups, we used the “COSG” R package (v0.9.0) to conduct differential analysis. The top 100 differential genes were screened and fed into the “clusterProfiler” R package (v4.12.0) for KEGG pathway enrichment. To explore the pathway of the target gene's function, the KEGG biological pathway analysis was conducted. Visualisation was carried out with the “enrichplot” R package (v1.24.0).

### 2.12 Molecular Docking

The study obtained the 2D structure of curcumin from the PubChem database and the PDB structure of Crispld2 from the UniProt database. Molecular docking was performed using curcumin as the ligand and the molecular target as the protein receptor. The results were analysed based on the binding stability and visualised using the CB‐DOCK2 website.

### 2.13 Cellular Thermal Shift Assay (CETSA)

Lysates of cells treated with curcumin or DMSO at 37°C for 4 h were collected. Each sample was heated at 45°C–60°C for 3 min, followed by cooling at room temperature for 3 min. After centrifugation at 12 000 rpm for 20 min at 4°C, the supernatants were boiled in loading buffer for Western blot (WB). Antibody information is provided in Table S1.

### 2.14 qRT‐PCR

Total RNA was isolated with TRIzol reagent (Invitrogen, USA), quantified for concentration and purity on a micro‐spectrophotometer (Thermo Fisher, USA), and reverse‐transcribed with PrimeScript RT Reagent Kit (Takara, Japan). qRT‐PCR was run with SYBR Premix Ex Taq II (Takara, Japan) on an ABI 7500 system (Applied Biosystems, USA). Relative expression was calculated with the 2^−ΔΔCt^ method, normalised to the internal reference gene GAPDH. Primer sequences were: Crispld2: F: AGGATTTGGACTGCTACACG; R: AGTAGGAAGGTTCGTCTTTGC. GAPDH: F: ACAACTTTGGTATCGTGGAAGG; R: GCCATCACGCCACAGTTTC.

### 2.15 Western Blot (WB)

Proteins were isolated from liver tissues or LX‐2 cells using RIPA buffer (Beyotime, China) and determined for concentrations with a BCA kit (Solarbio, China). Equal lysates were resolved by SDS‐PAGE, electro‐transferred to PVDF membranes (Beyotime, China), and blocked with 5% non‐fat milk. Membranes were probed overnight at 4°C with primary antibodies, then with HRP‐conjugated secondary antibodies. Signals were developed with ECL reagent (Beyotime, China). Band intensities were analysed by ImageJ (NIH, USA). Antibody information is listed in Table S1.

### 2.16 CCK‐8

Cells were seeded at 2 × 10^3^ cells/well in 96‐well plates. Following the indicated treatments, 10 μL CCK‐8 reagent (Beyotime, China) was added and cultured for 2 h at 37°C. OD_450 nm_ at each well was read on a microplate reader (Bio‐Rad, USA).

### 2.17 Flow Cytometry

Cells from each group were washed, plated at 3 × 10^5^ cells/well in 6‐well plates, and stained with 5 μL of Annexin V‐FITC and 5 μL of PI solution (Beyotime, China) for 15 min at room temperature protected from light. Apoptotic rates were immediately quantified on a FACSCalibur flow cytometer (BD Biosciences, USA).

### 2.18 TUNEL Staining

Paraffin‐embedded liver sections were processed for TUNEL staining with a commercial kit (Beyotime, China). After rehydration with graded ethanol, slides were treated with proteinase K solution at 37°C for 25 min. Sections were equilibrated in the supplied buffer, incubated with TUNEL reaction mixture for 60 min, protected from light, and counterstained with DAPI (Thermo Fisher, USA) for 30 min under light‐protected conditions. Following dehydration and clearing, coverslips were mounted. Apoptotic nuclei were observed under a fluorescence microscope (Olympus, Japan).

### 2.19 Co‐Immunoprecipitation (Co‐IP)

Cells were lysed in pre‐chilled RIPA buffer (Beyotime, China) at 4°C for 30 min and centrifuged (4°C). Supernatants were incubated overnight at 4°C with anti‐Crispld2 or control IgG and Protein A/G magnetic beads (MCE, USA). Beads were washed three times with buffer to remove unbound proteins. Immunoprecipitated Crispld2, PI3K, and AKT were detected by WB (antibodies: Table S1).

### 2.20 Statistical Analysis

Statistical analysis was performed using SPSS 26.0 (IBM, USA) and GraphPad Prism 8.0 (GraphPad, USA). All quantitative data are presented as mean ± standard deviation (SD). Comparisons between two groups were assessed by the unpaired two‐tailed Student's *t*‐test. Multiple group comparisons were analysed by one‐way ANOVA followed by Tukey's post hoc test. A *p*‐value < 0.05 was considered statistically significant.

## 3 Results

### 3.1 Curcumin Alleviates CCl_4_ ‐Induced Liver Injury and Fibrosis in Mice

**FIGURE 1:**
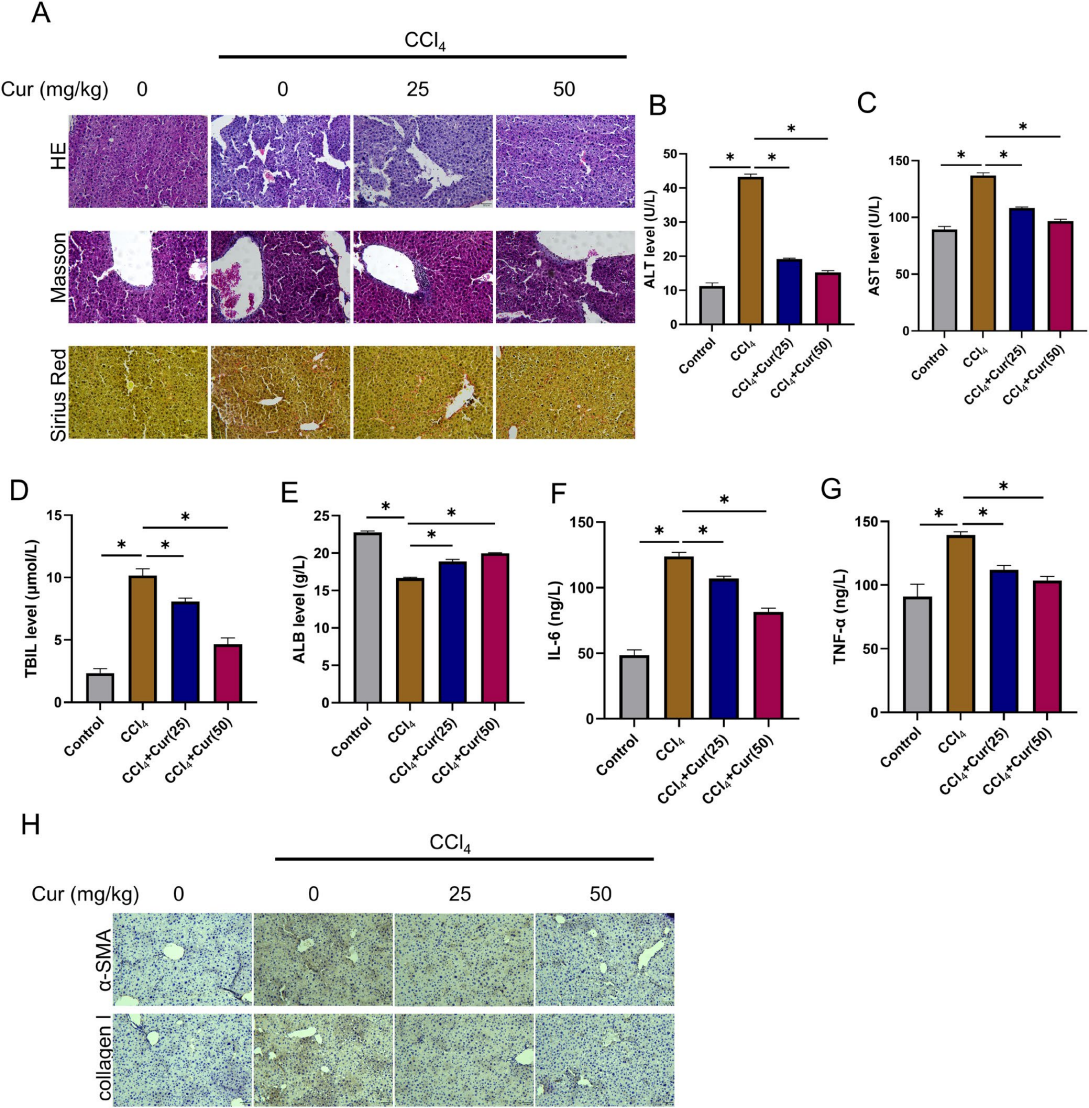
Curcumin alleviates CCl_4_‐induced liver injury and fibrosis in mice. Animal groups: Control, CCl_4_, CCl_4_ + Cur (25 mg/kg), CCl_4_ + Cur (50 mg/kg); *n* = 5. (A) Representative micrographs of H&E, Masson and Sirius‐Red staining. (B‐E) Serum levels of ALT (B), AST (C), TBIL (D) and ALB (E). (F, G) Serum IL‐6 (F) and TNF‐α (G) measured by ELISA. (H) IHC examination of the levels of fibrotic proteins (α‐SMA and collagen I) in mouse liver tissues. *p < 0.05.

To test curcumin's protective effect, mice were injected intraperitoneally with CCl_4_ to establish HF mouse models. Histological staining revealed that CCl_4_ caused inflammatory‐cell infiltration, hepatocyte steatosis, collagen deposition, mild centrilobular venous wall thickening, and fibroplastic proliferation. Curcumin dose‐dependently reversed these pathological changes, indicating a potent protective capacity against liver injury (Figure 1A). Serum markers of liver injury were next quantified. After CCl_4_ treatment, ALT, TBIL, and AST levels were significantly elevated, while ALB levels were decreased. Curcumin treatment significantly reversed these parameters (Figure 1B–E). Inflammation is a critical precursor to HF in chronic liver diseases. We next measured IL‐6 and TNF‐α by ELISA. As shown in Figure 1F,G, curcumin lowered both cytokines in a dose‐dependent manner. IHC further showed that CCl_4_‐induced increases in fibrotic proteins (α‐SMA and type I collagen) were mitigated by curcumin, with the 50 mg/kg dose exhibiting superior efficacy (Figure 1H). Collectively, these in vivo data demonstrated that curcumin dose‐dependently mitigated liver injury and fibrosis induced by CCl_4_.

### 3.2 scRNA‐Seq Elucidates the Mechanism of Curcumin in Alleviating CCl4‐Induced HF in Mice

**FIGURE 2:**
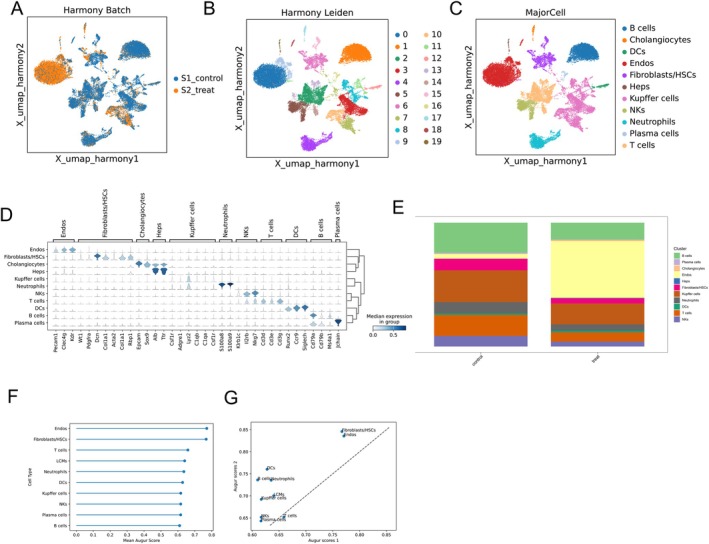
scRNA‐seq displays curcumin action in HF cell types. (A) Cell‐type annotations before and after curcumin. (B) UMAP showing cell annotations. (C) UMAP showing cell types. (D) Marker genes for cell type annotation. € Proportions of each cell type. (F) Ranking of Augur scores for each cell type. (G) Correlation analysis of perturbation scores for each cell type.

To delineate how curcumin attenuated CCl_4_‐induced mouse HF, we performed scRNA‐seq on livers harvested from HF mice with or without curcumin treatment. After stringent quality control (Figure S1A–D), 20 distinct clusters were resolved by UMAP (Figure 2A,B). Expression of marker genes was used to annotate cell types (Figure 2C), yielding 11 major types, including B cells, cholangiocytes, dendritic cells (DCs), endothelial cells (Endos), and fibroblasts/HSCs, etc. (Figure 2D). Comparative profiling across different samples revealed that after curcumin treatment, the levels of fibroblasts/HSCs, NKs, T cells, etc., all showed varying degrees of decrease (Figure 2E and S1E). To systematically evaluate the priority of curcumin treatment effects on various cell subsets in the liver fibrosis microenvironment, we established a perturbation model based on transcriptomic data and displayed the Augur score for each cell type. A higher Augur score indicates a greater impact of the perturbation on that cell type. Fibroblasts/HSCs and Endos were found to have the highest Augur scores, suggesting them as the cell populations most sensitive to curcumin intervention (Figure 2F,G). Collectively, the scRNA‐seq profiles of curcumin affecting HF identified fibroblasts/HSCs as the major perturbed cell type, whose expression was significantly lowered by curcumin treatment.

**FIGURE 3:**
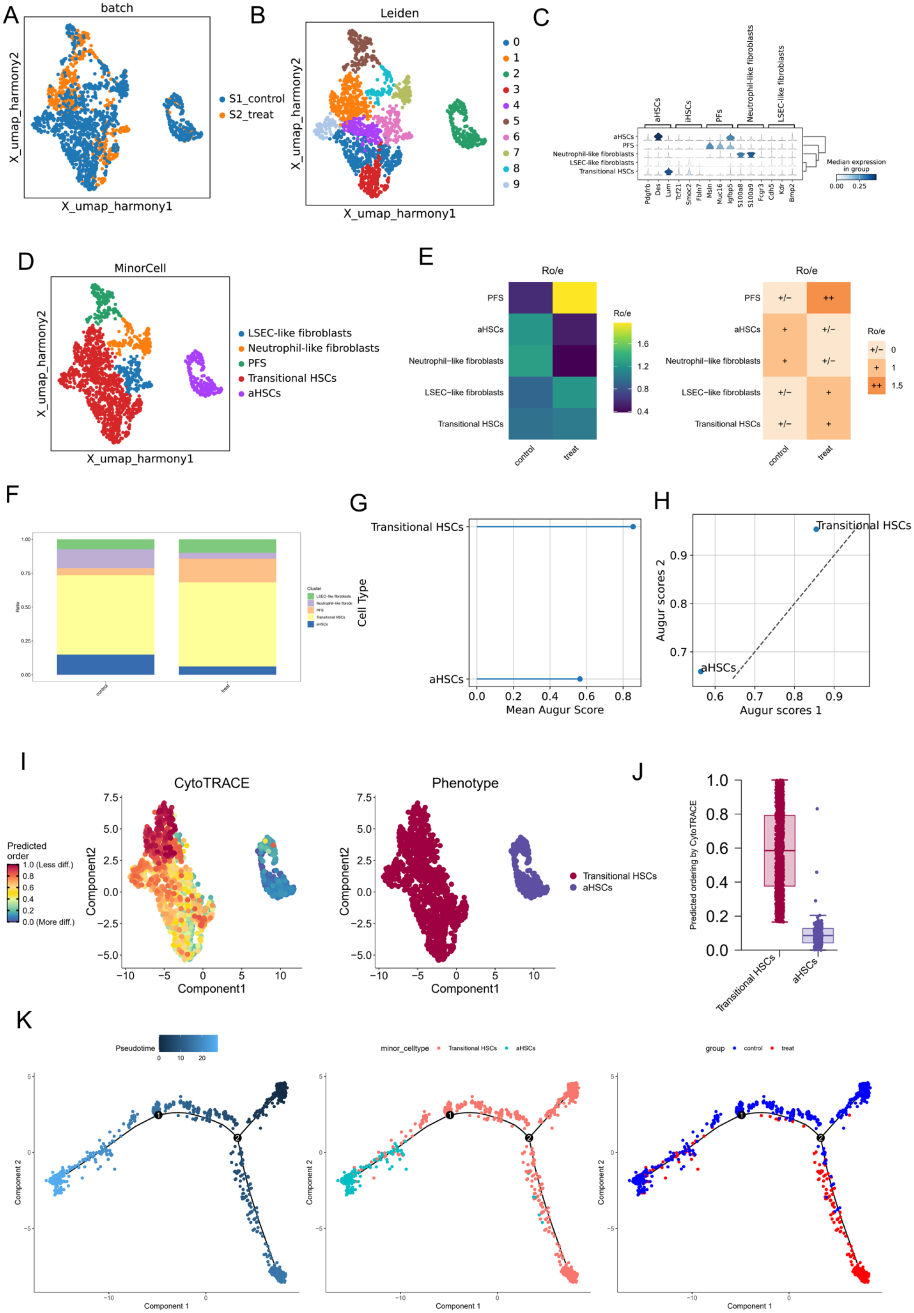
scRNA‐seq analysis of the effect of curcumin on fibroblast/HSCs. (A) Annotation of fibroblast/HSCs before and after curcumin treatment. (B) UMAP showing cell annotations of fibroblast/HSCs. (C) Marker genes annotating the subtypes of fibroblast/HSCs. (D) UMAP showing cell annotations of the subtypes of fibroblast/HSCs. (E) Heatmap showing the changes in the distribution of fibroblast/HSCs. (F) Proportions of the subtypes of fibroblast/HSCs. (G, H) Prediction of perturbed cell types. (I) UMAP showing the differentiation of fibroblast/HSC subtypes. (J) Differentiation potential scores of fibroblast/HSC subtypes. (K) Pseudotime trajectory showing the differentiation of fibroblast/HSC subtypes.

We next sub‐clustered the fibroblast/HSC type (Figure 3A,B) and, using established marker genes, resolved five subtypes: aHSCs, proliferative fibroblasts (PFS), neutrophil‐like fibroblasts, LSEC‐like fibroblasts and transitional HSCs (Figure 3C,D). Among them, aHSCs, as the core effector cells of liver fibrosis, highly express Des, Lum, etc., driving fibrosis through collagen secretion and enhanced cell contraction. The PFS subset highly expresses Msln, Muc16, etc., promoting fibrosis by regulating HSC activation and participating in liver tissue microenvironment remodelling. Neutrophil‐like fibroblasts highly express S100A8 and S100A9, accelerating fibrosis by chemotactically recruiting inflammatory cells and activating inflammatory pathways. Comparing the expression of each subtype, we revealed that after curcumin treatment, the proportions of aHSCs and neutrophil‐like fibroblasts were significantly decreased, whereas PFS, LSEC‐like fibroblasts and transitional HSCs expanded (Figure 3E,F). Perturbation modelling identified aHSCs and transitional HSCs as the most affected subtypes (Figure 3G,H). Subsequently, the differentiation potential of these two subtypes was analysed using CytoTRACE and Phenotype methods. It showed that aHSCs displayed lower differentiation potential than transitional HSCs, consistent with a post‐differentiation or quasi‐quiescent state (Figure 3I,J). Pseudotime trajectories confirmed that transitional HSCs localised to the early stage and aHSCs to the late stage of the differentiation path (Figure 3K). In conclusion, aHSCs were reduced after curcumin treatment and appeared to concentrate primarily at the late stage of differentiation. Of note, scRNA‐seq was performed on model and curcumin‐treated groups, without a normal control group, as the study aimed to elucidate the therapeutic mechanisms of curcumin in reversing established fibrosis by comparing pathological microenvironment changes before and after treatment. A complete dynamic landscape encompassing normal, disease, and drug intervention states will be addressed in future studies.

### 3.3 Curcumin Restrains HSC Activation by Targeting Crispld2

**FIGURE 4:**
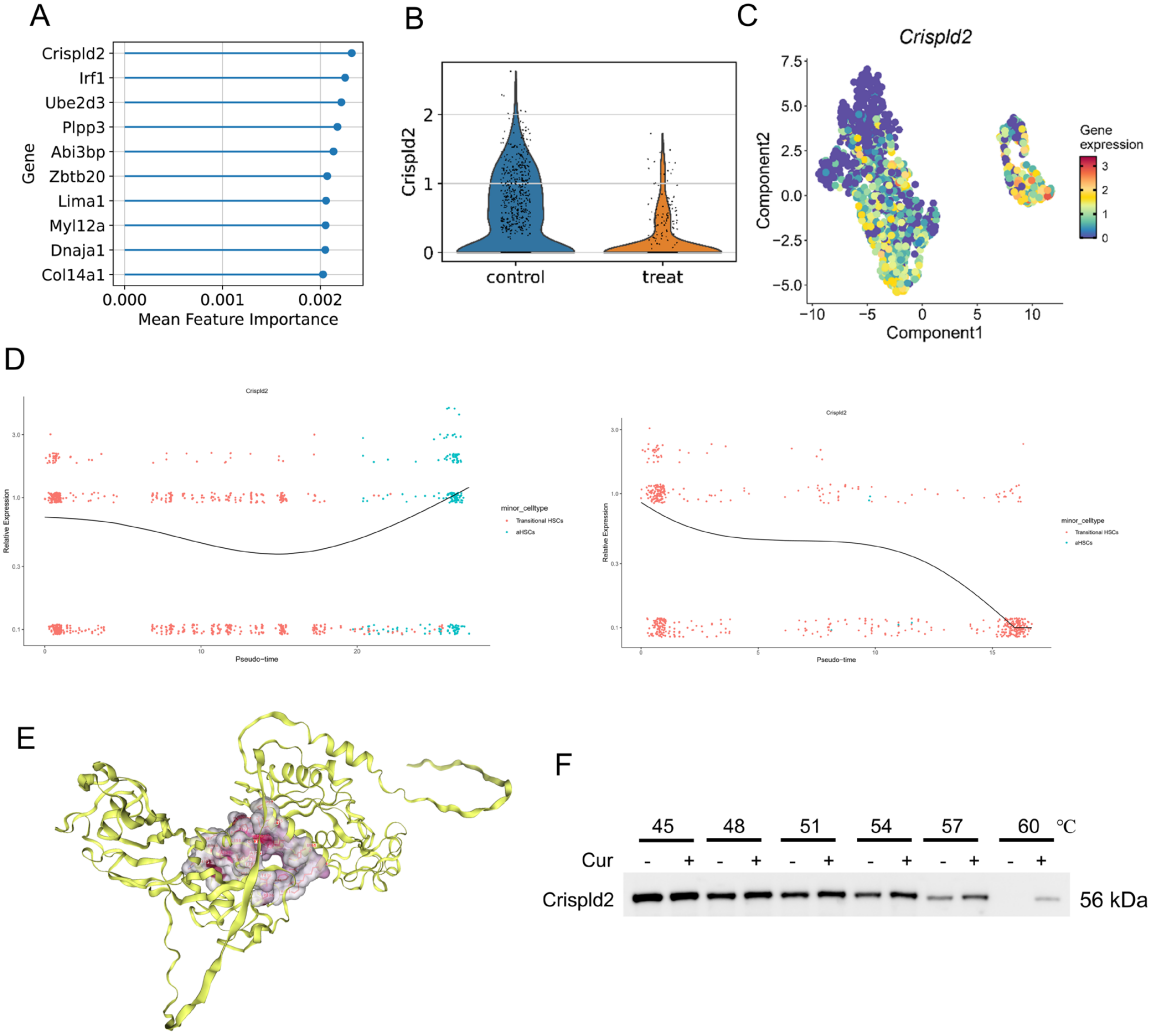
scRNA‐seq analysis of curcumin‐mediated regulation of Crispld2. (A) Ranking of feature genes contributing to perturbation response. (B) Expression of Crispld2 in transitional HSCs. (C) UMAP visualisation of Crispld2 expression during HSC differentiation. (D) Expression trajectories of Crispld2 in fibroblasts/HSCs in the Control (left) and Treatment (right) groups. (E) Molecular docking of curcumin with Crispld2. (F) CETSA validation of the binding interaction between curcumin and Crispld2.

Building on the above analysis that curcumin treatment reduced the number of aHSCs, we next predicted the key signature genes of perturbed genes and identified Crispld2 as a top candidate (Figure 4A). Its expression in transitional HSCs showed a significant decrease after curcumin exposure (Figure 4B,C). We next traced Crispld2 expression along the HSC‐differentiation trajectory. As shown in Figure 4D, curcumin treatment altered both the expression trends and the cellular distribution pattern of Crispld2 in the pseudotime differentiation of transitional HSCs and aHSCs. Next, molecular docking simulations preliminarily confirmed the direct interaction between curcumin and the Crispld2 protein, showing that binding stability is primarily maintained through hydrophobic interactions and hydrogen bonds (Figure 4E). CETSA further validated intracellular binding, revealing that curcumin treatment significantly enhanced the thermal stability of the Crispld2 protein (Figure 4F), confirming targeted binding between curcumin and the Crispld2 protein. Collectively, these findings reveal that curcumin may govern HSC fate by modulating Crispld2.

**FIGURE 5:**
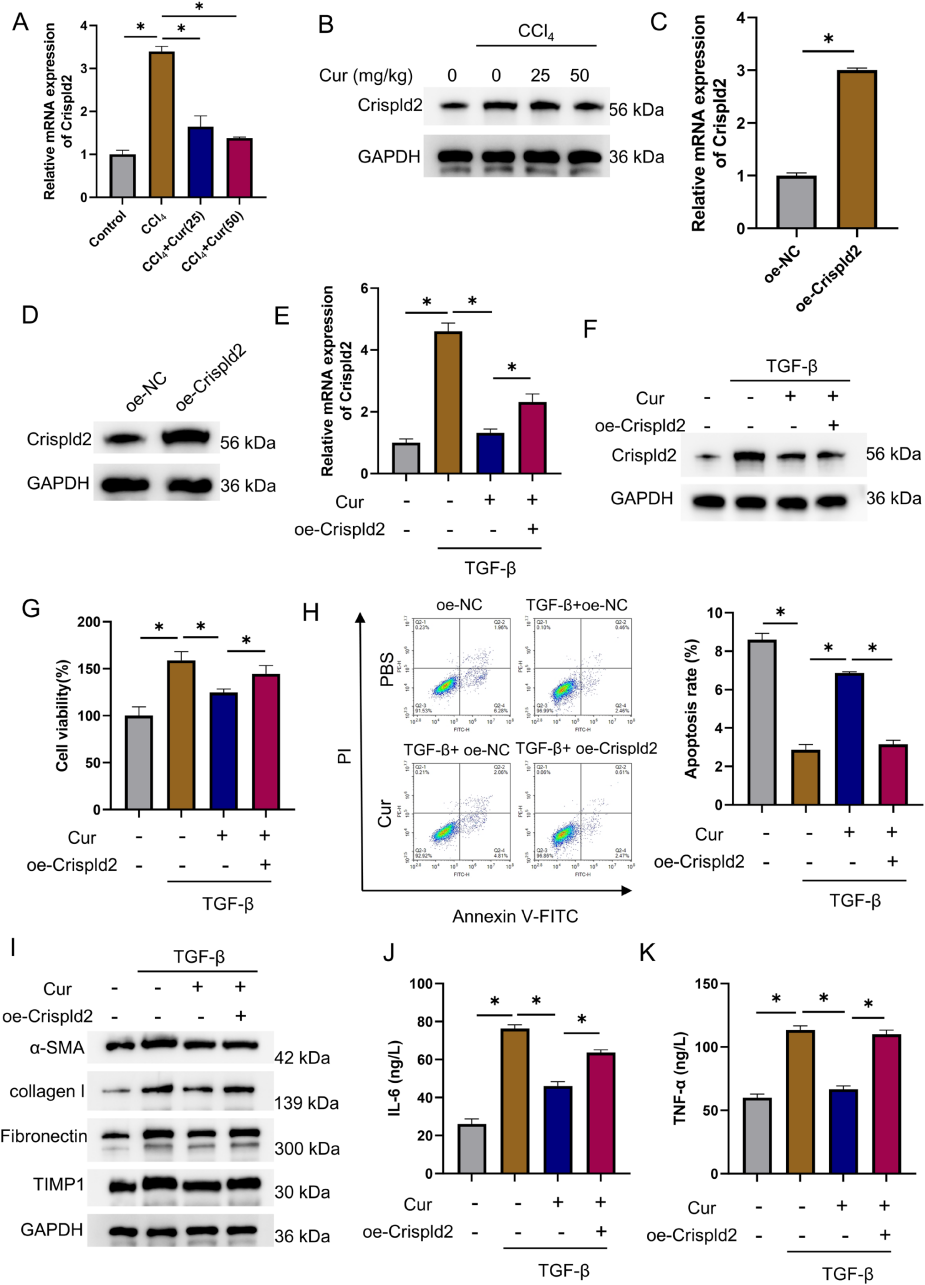
Curcumin suppresses HSC activation by targeting Crispld2. Animal groups: Control, CCl_4_, CCl_4_ + Cur (25 mg/kg), CCl_4_ + Cur (50 mg/kg); *n* = 5. (A) QRT‐PCR measuring the mRNA level of Crispld2. (B) WB determining the protein level of Crispld2. Groupings of LX‐2 cells: Oe‐NC, oe‐Crispld2. (C) QRT‐PCR verifying transfection efficiency. (D) WB confirming transfection efficiency. Groupings of LX‐2 cells with curcumin treatment: Control, TGF‐β + DMSO + oe‐NC, TGF‐β + Cur + oe‐NC, and TGF‐β + Cur + oe‐Crispld2. (E) QRT‐PCR detecting the mRNA levels of Crispld2. (F) WB examining the protein levels of Crispld2. (G) CCK‐8 assay assessing cell viability. (H) Flow cytometry measuring apoptosis. (I) WB detecting the expression of fibrotic proteins α‐SMA, collagen I, fibronectin, and TIMP1. (J, K) ELISA measuring the levels of inflammatory cytokines IL‐6 (J) and TNF‐α (K). *p < 0.05.

We subsequently examined Crispld2 expression in mouse livers by qRT‐PCR and WB. Results showed that curcumin suppressed Crispld2 dose‐dependently, with higher doses imposing stronger suppressive effects (Figure 5A,B). To further dissect how curcumin governed HSC activation, we established Crispld2‐overexpressing or Crispld2‐knockdown LX‐2 human HSCs. Efficient over‐expression was confirmed by qRT‐PCR and WB (Figure 5C,D S2A,B). Next, aHSCs were induced by exposing LX‐2 cells to 10 ng/mL TGF‐β and treated with curcumin. Experimental groups were: Control, TGF‐β + DMSO + oe‐NC, TGF‐β + Cur + oe‐NC, and TGF‐β + Cur + oe‐Crispld2. qRT‐PCR and WB revealed that curcumin suppressed the up‐regulation of Crispld2 induced by HSC activation, whereas overexpressing Crispld2 fully restored its expression, and Crispld2 knockdown resulted in a further reduction (Figure 5E,F S2C,D).

CCK‐8 and flow cytometry next showed that curcumin reduced the viability of aHSCs and increased their apoptosis, whereas Crispld2 knockdown further enhanced these effects. Both effects were significantly reversed by Crispld2 overexpression (Figure 5G,H S2E,F). WB was performed to measure the levels of fibrotic proteins and evaluate HSC fibrosis. We found that curcumin significantly down‐regulated α‐SMA, collagen I, fibronectin, and matrix metalloproteinase 1 (TIMP1), whereas Crispld2 knockdown enhanced the inhibitory effects of curcumin (Figure 5I S2G). Finally, ELISA revealed that Crispld2 up‐regulation fully rescued the curcumin‐imposed suppression of IL‐6 and TNF‐α; similarly, Crispld2 knockdown enhanced the inhibitory effects of curcumin on these inflammatory cytokines (Figure 5J,K S2H,I). Collectively, curcumin treatment suppressed HSC activation by targeting Crispld2.

### 3.4 Curcumin Treatment Suppresses HSC Activation by Modulating the Crispld2 to Mediate the PI3K/AKT Axis

**FIGURE 6:**
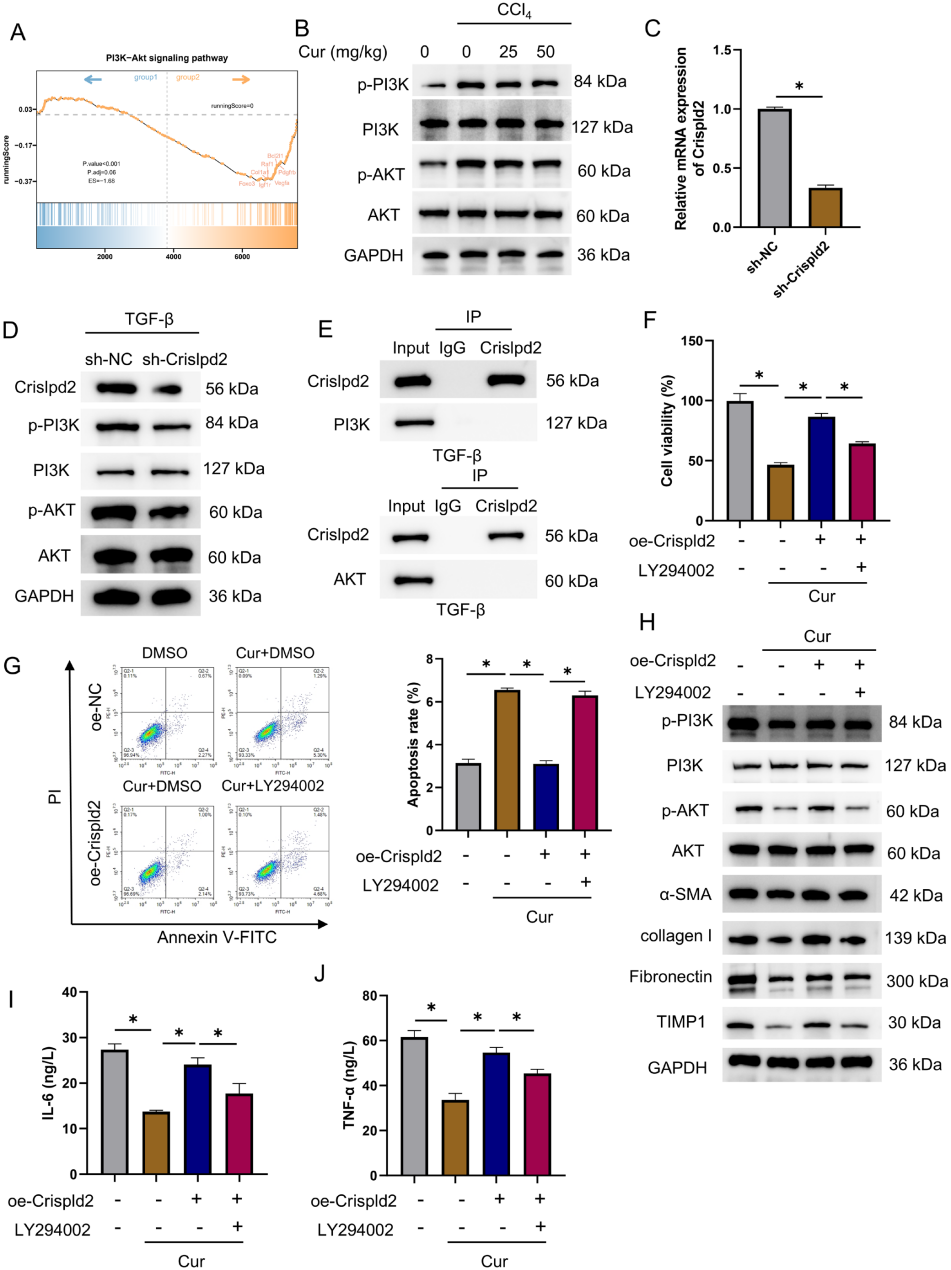
Curcumin treatment suppresses HSC activation by modulating the Crispld2 to mediate the PI3K/AKT axis. (A) KEGG enrichment of Crispld2 in the transitional HSC treatment group. Animal groups: Control, CCl_4_, CCl_4_ + Cur (25 mg/kg), CCl_4_ + Cur (50 mg/kg); *n* = 5. (B) WB assessing the protein levels of p‐PI3K, PI3K, p‐AKT, and AKT. Groups of LX‐2 cells treated by TGF‐β: Sh‐NC, sh‐Crispld2. (C) QRT‐PCR quantification of Crispld2 mRNA. (D) WB analysis of Crispld2, p‐PI3K, PI3K, p‐AKT, AKT. Experimental groups of LX‐2 cells with Crispld2 overexpression induced by TGF‐β, following curcumin or LY294002 treatment: Control, Cur + oe‐NC, Cur + oe‐Crispld2, Cur + oe‐Crispld2 + LY294002. (**E) Co‐IP** assays confirmed the interaction of CRISPLD2 with PI3K and AKT. (F) CCK‐8 assessing cell viability. (G) Flow cytometry assessing apoptosis. (H) WB for p‐PI3K, PI3K, p‐AKT, AKT and fibrotic proteins (α‐SMA, collagen I, fibronectin, TIMP1). (I, J) ELISA detection of inflammatory cytokines IL‐6 (I) and TNF‐α (J). *p < 0.05.

According to scRNA‐seq, Crispld2 in the transitional HSCs treatment group showed significant enrichment in the PI3K/AKT pathway (Figure 6A). WB of mouse liver tissues examined the protein expression in the PI3K/AKT pathway and confirmed that curcumin dose‐dependently decreased p‐PI3K and p‐AKT (Figure 6B). We subsequently generated LX‐2 cells with Crispld2 knockdown. Knockdown efficiency was verified by qRT‐PCR and WB (Figure 6C,D). After the activation induced by TGF‐β, the examination of protein expression in the PI3K/AKT pathway showed that Crispld2 knockdown suppressed the activation of the PI3K/AKT pathway, which further confirmed the regulatory function of Crispld2 on the pathway (Figure 6D). We next explored the interaction between Crispld2 and the PI3K/AKT pathway. Co‐IP revealed no direct binding of Crispld2 with the PI3K or AKT protein (Figure 6E). We infer that Crispld2, as a secreted protein, acts extracellularly by binding unknown membrane receptors to trigger downstream signalling cascades, rather than forming a physical complex with PI3K/AKT intracellularly.

Previous work has reported that the PI3K/AKT signalling pathway critically drives HF [20]. We therefore hypothesised that curcumin suppressed HSC activation by modulating Crispld2 to regulate the PI3K/AKT axis. To test this, Crispld2 was overexpressed in curcumin‐treated aHSCs, and the PI3K/AKT inhibitor LY294002 (MCE, USA) was added for a rescue experiment. CCK‐8 and flow cytometry showed that Crispld2 overexpression significantly reversed the curcumin‐induced loss of viability and the increase in cell death. These effects were reversed when LY294002 was present (Figure 6F,G). Subsequently, the expression of the PI3K/AKT pathway and fibrotic proteins was measured by WB. As expected, Crispld2 overexpression, on top of curcumin treatment, significantly activated the PI3K/AKT axis and up‐regulated fibrosis of HSCs. These phenotypes were suppressed to varying degrees upon the addition of LY294002 (Figure 6H). Finally, Crispld2 overexpression also elevated pro‐inflammatory cytokine levels in curcumin‐treated aHSCs, and this escalation was suppressed upon the PI3K/AKT blockade (Figure 6I,J). Collectively, these data indicated that curcumin restrained HSC activation by regulating Crispld2‐mediated PI3K/AKT signalling.

### 3.5 Animal Experiments Confirm That Curcumin Treatment Alleviates CCl_4_ ‐Induced Liver Injury and Fibrosis in Mice by Modulating the Crispld2/PI3K/AKT Axis

**FIGURE 7:**
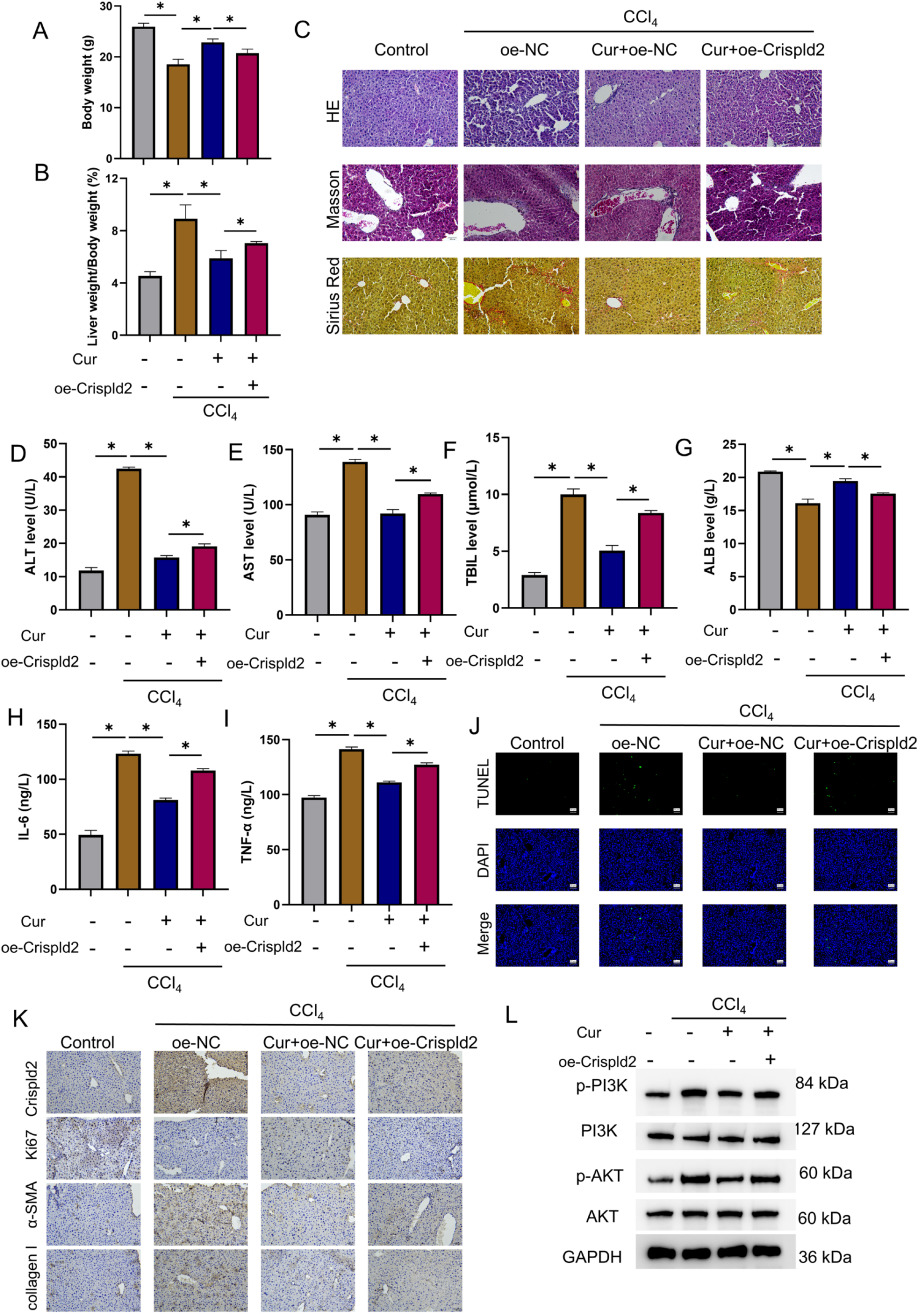
Animal experiments confirm that curcumin treatment alleviates CCl4‐induced liver injury and fibrosis in mice by modulating the Crispld2/PI3K/AKT axis. Groups: Control, CCl_4_, CCl_4_ + Cur + oe‐NC, CCl_4_ + Cur + oe‐Crispld2. Curcumin dose: 50 mg/kg; *n* = 5. (A) Body‐weight changes of mice. (B) Liver‐to‐body weight ratio of mice. (C) Representative H&E, Masson and Sirius‐red staining of liver tissues. (D‐G) Serum levels of ALT (D), AST (E), TBIL (F) and ALB (G). (H, I) Serum inflammatory cytokines IL‐6 (H) and TNF‐α (I) were measured by ELISA. (J) TUNEL assay for apoptosis in mouse liver tissues. (K) IHC for Crispld2, Ki67, and fibrotic proteins (α‐SMA and collagen I) in liver tissues. (L) WB analysis of the protein levels of p‐PI3K, PI3K, p‐AKT and AKT. *p < 0.05.

To corroborate the in vitro results, we employed a mouse model of CCl_4_‐induced HF. Curcumin was administered, and Crispld2 was overexpressed via a lentiviral vector. The experimental groups were: Control, CCl4 + DMSO + oe‐NC, CCl_4_ + Cur + oe‐NC, and CCl_4_ + Cur + oe‐Crispld2. Curcumin treatment reduced body weight and increased the liver‐to‐body weight ratio in fibrotic mice, which was markedly reversed when Crispld2 was up‐regulated (Figure 7A,B). Similarly, Crispld2 overexpression reversed curcumin‐induced improvements in fibrotic pathology, liver function, and the suppression of pro‐inflammatory cytokines (Figure 7C–I). TUNEL assay of liver tissues showed that curcumin treatment inhibited apoptosis by regulating Crispld2 (Figure 7J). IHC corroborated these results: Curcumin lowered hepatic expression of Crispld2, α‐SMA, and collagen I, raised Ki67 levels in mice with HF, and all of these changes were reversed by Crispld2 overexpression (Figure 7K). WB confirmed the in vitro data, revealing that curcumin suppressed the activation of the PI3K/AKT pathway by regulating Crispld2 expression (Figure 7L). Collectively, the animal study established that curcumin alleviated mouse HF through the Crispld2/PI3K/AKT axis.

## 4 Discussion

It is well‐established that dysregulated ECM synthesis and degradation drive HF and eventual cirrhosis [21]. The myofibroblasts that produce ECM in the liver mainly originate from HSCs. Therefore, targeted drug intervention on HSCs could be an effective therapeutic strategy for HF [22]. In this study, we confirmed that curcumin was protective against inflammation, CCl_4_‐induced liver injury, and HF. By integrating scRNA‐seq with complementary cellular and animal experiments, we further identified Crispld2 as a previously unrecognised curcumin target that mediated the PI3K/AKT signalling pathway and thereby influenced the activation and apoptosis of HSCs. These findings provided a new mechanistic explanation for the antifibrotic activity of curcumin.

A mouse model was established to investigate the role of curcumin in HF. It was confirmed that curcumin reduced hepatic expression of α‐SMA and collagen I, while concurrently ameliorating structural damage and restoring liver function. This is consistent with the report by Li et al. [23], regarding the regulatory roles of curcumin in early and late‐stage HF. In addition, scRNA‐seq resolved the dynamic cell changes in the fibrosis process induced by CCl_4_ in mouse livers and revealed that curcumin decreased the numbers of aHSCs while expanding a transitional HSC population. This observation aligns with Erika et al. [24], who showed that curcumin blocks HSC activation by interrupting the TGF‐β/Smad2 signalling. aHSCs are the principal executors of HF, whose reduction translates into less ECM synthesis and lower levels of α‐SMA, collagen I, and other fibrogenic proteins, ultimately slowing fibrosis progression [25]. In our research, curcumin suppressed the activation of HSCs and their anti‐apoptotic capability. Meanwhile, it was confirmed that it reduced the release of pro‐inflammatory cytokines and down‐regulated fibrotic proteins. This evidence collectively confirmed that curcumin may play a pivotal role in suppressing HSC activation and alleviating HF progression. However, a limitation of our scRNA‐seq is the absence of normal liver tissue as a control, which restricts a comprehensive analysis of cell lineage changes during the transition from normal to fibrotic states. Future studies incorporating normal control samples will further refine the cellular dynamics model of liver fibrosis development and reversal.

To dissect the molecular circuitry through which curcumin restrained HSC activation, we constructed a perturbation model and screened for signature genes perturbed during HSC differentiation, identifying Crispld2. Crispld2 belongs to the secreted protein family and has been previously reported to participate in inflammatory response regulation [26]. However, the functional role of Crispld2 in liver diseases, particularly in the process of liver fibrosis, remains unexplored. Liver fibrosis is characterised by persistent HSC activation and excessive ECM deposition, with the inflammatory microenvironment serving as a key driver of HSC activation and fibrosis progression. Given the established role of Crispld2 in inflammatory regulation, we hypothesised that it may influence liver fibrosis progression by mediating HSC activation and modulating the release of inflammatory factors. Through bidirectional experimental strategies of overexpression and knockdown, we confirmed the high expression of Crispld2 in aHSCs and its downregulation following curcumin treatment, which led to a significant reduction in HSC activity and suppression of fibrosis‐related proteins and inflammatory factors. Previous work has primarily focused on the role of Crispld2, a newly recognised target, in embryonic development and tumour metastasis [27]. This study extended its functional relevance to the field of HF, revealing its critical involvement in HSC activation and offering important theoretical insights. However, the underlying mechanisms through which Crispld2 regulated HSCs remain to be elucidated.

Several signalling cascades, such as Wnt/β‐catenin [28], Hippo [29] and p38 MAPK [30] have been noticed in the activation of HSCs and the initiation and progression of HF. Previous studies have reported activation of the HIF‐1α and ERK pathways in the CCl₄‐induced liver fibrosis rat model [31]. Meanwhile, curcumin is known to inhibit HSC activation via multiple pathways, including TGF‐β/Smad and autophagy [14, 24]. Our scRNA‐seq revealed that Crispld2 was significantly enriched in the PI3K/AKT pathway within the transitional HSC treatment group. Although PI3K/AKT is established as a key node in liver fibrosis and HSC activation [7, 32], its upstream regulation, particularly the role of Crispld2, has remained unexplored. Through in vivo and in vitro experiments, we identified a novel regulatory axis: Curcumin inhibits HSC activation, fibrosis progression, and inflammatory cytokine release by regulating Crispld2 to suppress PI3K/AKT signalling in HSCs. This finding addresses a critical gap in understanding the Crispld2‐mediated PI3K/AKT pathway in liver fibrosis and offers a novel theoretical foundation for targeting HSC activation. Notably, while curcumin exerts therapeutic effects via PI3K/AKT in kidney disease [33], cancer [34], and neurodegenerative disorders [35], its specific molecular mechanism in liver fibrosis has not been fully elucidated. Given that TGF‐β/Smad, as a classic pro‐fibrotic pathway, primarily mediates ECM synthesis and HSC phenotype transformation [36], whereas HIF‐1α and ERK are more engaged in sustained HSC activation and autophagy under hypoxic stress [37, 38], we propose that the anti‐fibrotic action of curcumin involves a multi‐target network, with the Crispld2/PI3K/AKT axis potentially interacting with pathways. Reportedly, curcumin acts through direct inhibition of the TGF‐β/Smad2 pathway and activation of autophagy; in our study, it downregulated Crispld2 to suppress the PI3K/AKT axis, attenuating its cross‐activation with HIF‐1α/ERK and other pathways, forming a “multi‐pathway, multi‐node” network inhibitory effect. These findings expand the evidence for the multi‐target therapeutic effects of curcumin and, by uncovering the Crispld2/PI3K/AKT axis, offer a new direction for its clinical application in liver fibrosis treatment.

In summary, by integrating scRNA‐seq with cellular and animal assays, we demonstrate that curcumin inhibits the PI3K/AKT cascade by targeting Crispld2, consequently restraining HSC activation and alleviating HF. Several limitations warrant consideration. First, although curcumin regulates the PI3K/AKT pathway via Crispld2, no direct interaction exists between Crispld2 and PI3K/AKT proteins. Thus, the precise molecular mechanism underlying their crosstalk remains elusive. Given that Crispld2 is a secreted protein, it likely activates PI3K/AKT indirectly, potentially through binding to cell membrane receptors or modulating intracellular signalling cascades. Future studies employing mass spectrometry and related approaches will aim to identify the membrane receptors of Crispld2 and clarify the downstream bridging mechanism linking these receptors to PI3K/AKT signalling, unravelling the molecular network through which Crispld2 regulates HSC activation. Second, the therapeutic window and long‐term safety of curcumin require rigorous pharmacokinetic and toxicological evaluation in larger animal models. Finally, translating these findings to human HF necessitates validation using primary human HSCs and explant tissues. Future work will address these issues to establish a firmer mechanistic and translational basis for precision therapy of HF.

## 5 Conclusion

In summary, this study establishes a novel curcumin‐Crispld2‐PI3K/AKT regulatory axis governing HSC activation, providing both a mechanistic foundation for curcumin's anti‐fibrotic efficacy and a paradigm for targeting secreted protein regulators in fibrotic diseases. These findings position Crispld2 as a promising biomarker and therapeutic target for precision management of HF.

## Author Contributions

L.L., JT.Z. and YQ.S. designed and performed the experiments. L.L., JT.Z. and Y.W. performed the analysis and data curation. L.L. and CK.J. provided funding acquisition. L.L., Y.W., JT.Z. and CA.Y. performed writing – original draft. CK.J. and YQ.S. performed writing – editing. All the authors performed writing – review. All authors gave the final approval of the version to be published and agreed to be accountable for all aspects of the work.

## Funding

This work was supported by the Zhejiang Provincial Health Commission, Zhejiang Traditional Chinese Medicine Science and Technology Plan Project (Grant No. 2022ZB275) and the Construction Fund of Key Medical Disciplines of Hangzhou (Grant No. 2025HZGF05).

## Ethics Statement

This study protocol was reviewed and approved by the Animal Research Ethics Committee of the Second Affiliated Hospital, School of Medicine, Zhejiang University in China (No. 2025–273).

## Conflicts of Interest

The authors declare no conflicts of interest.

## Supporting information


**Figure S1:** Quality control of the scRNA‐seq data. (A) Violin plot showing the percentage of mitochondrial and ribosomal genes after filtering. (B) Correlation between nUMI and the number of genes detected. (C) Comparison of batch effect correction results across five methods: Harmony, scVI, scANVI, BBKNN, and Scanorama. (D) Results of the PCA with the top 30 principal components. € Heatmap illustrating the distribution changes across identified cell types.
**Figure S2:** Curcumin treatment inhibits HSC activation by regulating Crispld2. LX‐2 cell groups: sh‐NC and sh‐Crispld2. (A) qRT‐PCR validation of transfection efficiency; (B) WB validation of transfection efficiency. LX‐2 cells were treated with TGF‐β followed by curcumin, with groups: Control, TGF‐β + DMSO+sh‐NC, TGF‐β + Cur + sh‐NC, TGF‐β + Cur + sh‐Crispld2. (C) qRT‐PCR detection of Crispld2 mRNA levels; (D) WB detection of Crispld2 protein levels; (E) CCK‐8 assay for cell viability; (F) Flow cytometry for apoptosis; (G) WB detection of fibrosis‐related proteins α‐SMA, collagen I, fibronectin, and TIMP1; H‐I: ELISA detection of inflammatory cytokine levels of IL‐6 (H) and TNF‐α (I). *p < 0.05.
**Table S1:** Antibody information for assays.

## Data Availability

The raw single‐cell RNA sequencing data supporting the conclusions of this study have been deposited in the figshare database and are publicly available as of the date of publication. The dataset can be accessed via the following link: https://doi.org/10.6084/m9.figshare.30833288. This study did not generate any new code. Any other information required for reanalysing the data reported in this article can be obtained from the corresponding author.
