# Supplementary material for: Curcumin Targets Crispld2 to Suppress Hepatic Stellate Cell Activation via PI3K/AKT Pathway Inhibition in Hepatic Fibrosis
Source: Liver Int. 2026 May 13;46:e70696. doi: 10.1111/liv.70696 (PMC13172649; doi:10.1111/liv.70696)
Supplement: Supplementary file 1 — Figure S1: Quality control of the scRNA‐seq data. (A) Violin plot showing the percentage of mitochondrial and ribosomal genes after filtering. (B) Correlation between nUMI and the number of genes detected. (C) Comparison of batch effect correction results across five methods: Harmony, scVI, scANVI, BBKNN, and Scanorama. (D) Results of the PCA with the top 30 principal components. € Heatmap illustrating the distribution changes across identified cell types. Figure S2: Curcumin treatment inhibits HSC activation by regulating Crispld2. LX‐2 cell groups: sh‐NC and sh‐Crispld2. (A) qRT‐PCR validation of transfection efficiency; (B) WB validation of transfection efficiency. LX‐2 cells were treated with TGF‐β followed by curcumin, with groups: Control, TGF‐β + DMSO+sh‐NC, TGF‐β + Cur + sh‐NC, TGF‐β + Cur + sh‐Crispld2. (C) qRT‐PCR detection of Crispld2 mRNA levels; (D) WB detection of Crispld2 protein levels; (E) CCK‐8 assay for cell viability; (F) Flow cytometry for apoptosis; (G) WB detection of fibrosis‐related proteins α‐SMA, collagen I, fibronectin, and TIMP1; H‐I: ELISA detection of inflammatory cytokine levels of IL‐6 (H) and TNF‐α (I). *p < 0.05. Table S1: Antibody information for assays. [file LIV-46-0-s001.docx]

**Supplementary Table**

**Table S1. Antibody information for assays**

| Antibody | Cat. No. | Supplier | Assay |
| --- | --- | --- | --- |
| Mouse anti-Crispld2 | M080175 | Abmart, China | WB, IHC |
| Rabbit anti-α-SMA | A2235 | ABclonal, China | WB, IHC |
| Rabbit anti-collagen I | A24112 | ABclonal, China | WB, IHC |
| Rabbit anti-Fibronectin | A12977 | ABclonal, China | WB |
| Rabbit anti-TIMP1 | ab211926 | Abcam, UK | WB |
| Rabbit anti-p-PI3K | AF3241 | Affinity, China | WB |
| Rabbit anti-PI3K | ab302958 | Abcam, UK | WB |
| Rabbit anti-p-AKT | AP0637 | ABclonal, China | WB |
| Rabbit anti-AKT | A17909 | ABclonal, China | WB |
| Rabbit anti-KI67 | ab16667 | Abcam, UK | IHC |
| Rabbit anti-GAPDH | ab181602 | Abcam, UK | WB |
| Goat anti-Rabbit IgG H&L (HRP) | ab6721 | Abcam, UK | IHC, WB |
| Goat anti-Mouse IgG H&L (HRP) | ab205719 | Abcam, UK | IHC, WB |

**Supplementary Figure(Figure S1, S2)**

**
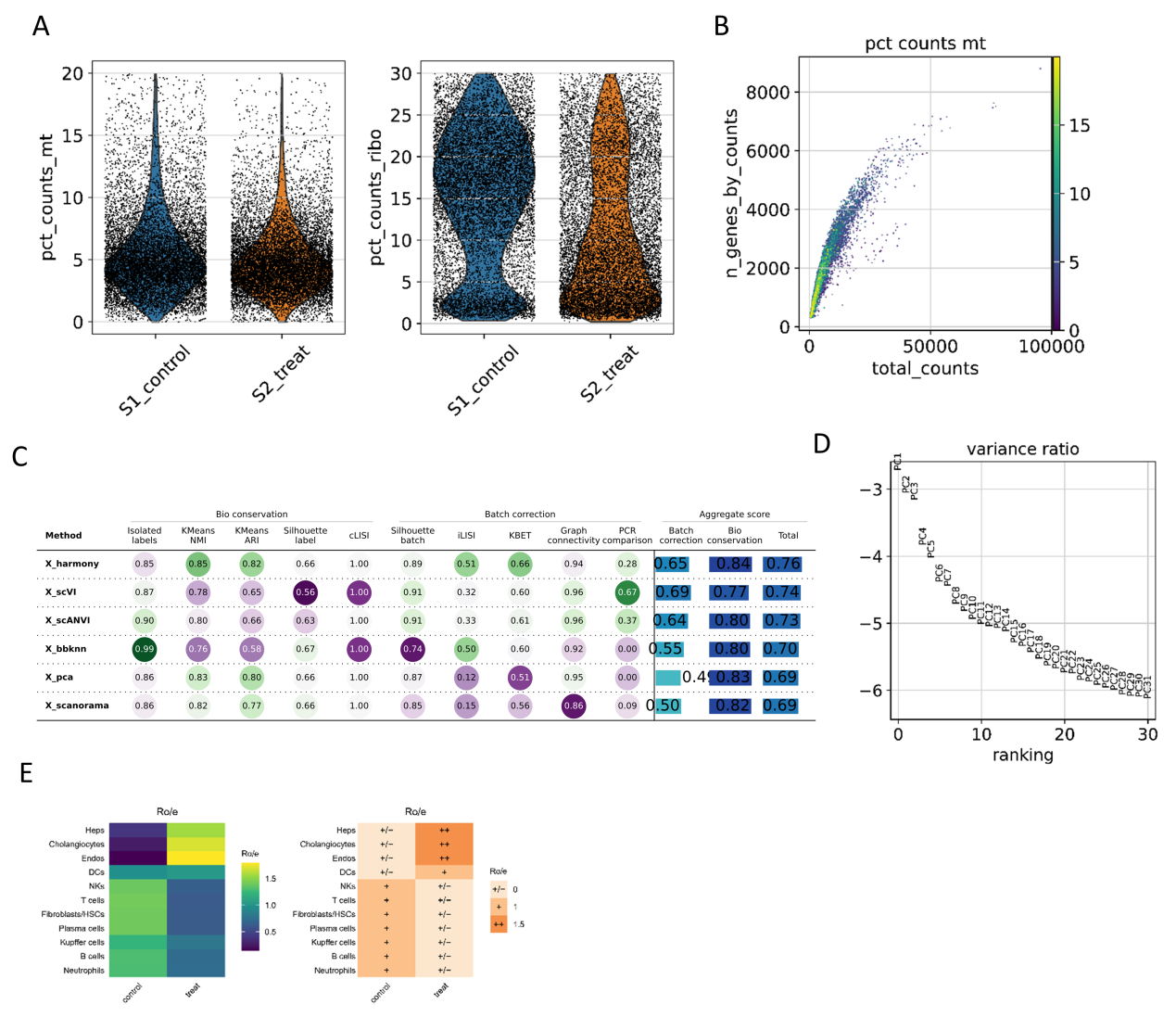
**

**Figure S1. Quality control of the scRNA-seq data. A:** Violin plot showing the percentage of mitochondrial and ribosomal genes after filtering. **B:** Correlation between nUMI and the number of genes detected. **C:** Comparison of batch effect correction results across five methods: Harmony, scVI, scANVI, BBKNN, and Scanorama. **D:** Results of the PCA with the top 30 principal components. **E:** Heatmap illustrating the distribution changes across identified cell types.


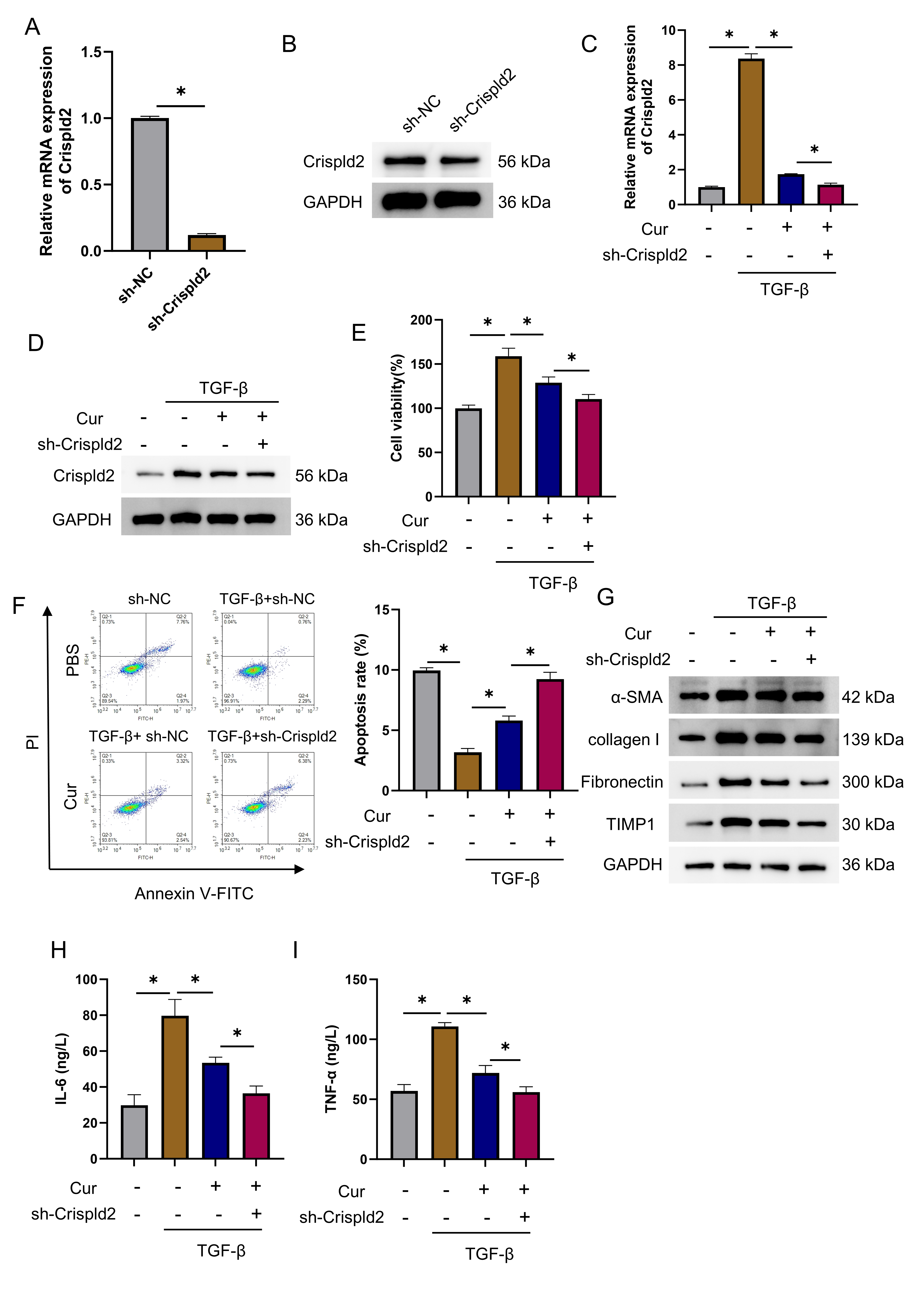


**Figure S2:** Curcumin treatment inhibits HSC activation by regulating Crispld2. LX-2 cell groups: sh-NC and sh-Crispld2. **A:** qRT-PCR validation of transfection efficiency; **B:** WB validation of transfection efficiency. LX-2 cells were treated with TGF-β followed by curcumin, with groups: Control, TGF-β+DMSO+sh-NC, TGF-β+Cur+sh-NC, TGF-β+Cur+sh-Crispld2. **C:** qRT-PCR detection of Crispld2 mRNA levels; **D:** WB detection of Crispld2 protein levels; **E:** CCK-8 assay for cell viability; **F:** Flow cytometry for apoptosis; **G:** WB detection of fibrosis-related proteins α-SMA, collagen I, fibronectin, and TIMP1; H-I: ELISA detection of inflammatory cytokine levels of IL-6 (H) and TNF-α (I). * P < 0.05.
